# Intergenerational Transmission of Reflective Functioning

**DOI:** 10.3389/fpsyg.2016.01903

**Published:** 2016-12-06

**Authors:** Anna M. Rosso, Cinzia Airaldi

**Affiliations:** Department of Education, University of GenoaGenova, Italy

**Keywords:** child reflective functioning, maternal mentalization, Child Attachment Interview, preadolescence, dismissing attachment model

## Abstract

The present study investigated whether, and to what extent, reflective functioning (RF) during preadolescence is associated with maternal attachment security and RF, and with the child’s attachment security. Thirty-nine mother–preadolescent child dyads from a non-clinical population participated in the study. Maternal and child RF were assessed by applying the Reflective Functioning Scale to the Adult Attachment Interview (AAI) and to the Child Attachment Interview transcripts. Children of mothers who showed a secure attachment model regarding the relationship with their parents during childhood reported higher levels of RF than the children of mothers who were classified as insecure on the AAI. Child RF was positively associated with maternal “Coherence of the Mind” on the AAI and negatively associated with maternal derogation of attachment. A strong, significant association was also found between child attachment security and child RF. Children who were rated as being more emotionally open, more able to balance positive and negative descriptions of their parents, more prone to support their assertions through examples, and more able to positively resolve conflicts with their parents showed higher RF. On the contrary, children who resorted to a higher extent to idealization and dismissal toward their parents showed a lesser degree of RF. Notably, a very strong association was found between the score on the “Overall coherence” subscale and the child’s ability to mentalize mixed-ambivalent mental states in the context of their family relationships. As expected, child and maternal RF resulted significantly positively correlated with each other. In particular, only maternal RF (and not maternal attachment security) predicted child RF, and only maternal ability to mentalize mixed-ambivalent mental states predicted the corresponding ability in the children.

## Introduction

The development of the human ability to understand the mental states of oneself and of others has been studied by philosophers (e.g., Brentano, 1924; Dennett, 1987; Fodor, 1987), cognitive and developmental psychologists (e.g., Baron-Cohen et al., 1985; Dunn, 1988; Gopnik and Astington, 1988), and neuroscientists (e.g., LeDoux, 1996). This ability is commonly referred to as “mentalization.” However, a growing body of evidence supports the notion that the construct of mentalization includes several components which are only partially correlated to each other (Fonagy et al., 2012). In addition, the term “mentalization” often refers to different constructs [e.g., theory of mind (ToM), mind-mindedness, emotional intelligence] which albeit partially overlapping, originated from different theoretical frameworks and were investigated by means of different experimental paradigms or tasks (Sharp and Fonagy, 2008).

In this study, we focused on reflective functioning (RF), a definite operationalization of mentalization that was suggested by Fonagy and Target (1997) in the context of the attachment theory. RF was defined as the ability to mentalize in the context of close, interpersonal relationships, thus allowing “to distinguish inner from outer reality, pretend from ‘real’ modes of functioning, intra-personal mental and emotional processes from interpersonal communications” (Fonagy et al., 1998). It promotes a more coherent sense of self as well as a better understanding of others, thereby making the individuals’ behavior meaningful and predictable. It is assumed that RF originates in the context of early attachment relationships and is promoted by a mentalizing mother who is able to treat her child as a being with a mind, and can keep her child’s feelings, desires as well as intentions in her own mind (Fonagy et al., 2002). Such a mentalizing mother helps the child to recognize, tolerate, and regulate his/her emotional experiences through her ability to represent them, through her gestures and actions, and later also by playing and talking in terms of mental states (Gergely and Watson, 1996; Meins et al., 2002).

Reflective functioning was initially assessed in adults by applying the Reflective Functioning Scale (RFS) to the Adult Attachment Interview (AAI) (Main and Goldwyn, 1998), a semi-structured interview which focuses on the subject’s attachment experiences with their parents during childhood. As will be further explained in more detail, some questions in the AAI (e.g., “Why did your parents behave as they did during your childhood?,” “Do you think your childhood experiences have an influence on who you are today?”) require RF, while others allow it. Based on the RFS, RF emerges when the interviewee shows that he/she is aware of the nature of mental states, an explicit effort to tease out mental states underlying behavior, the proneness to recognize developmental aspects of mental states or mental states in relation to the interviewer (Fonagy et al., 1998). The longitudinal London Parent–Child Project (Fonagy et al., 1991) found that mothers with higher RF (who were interviewed during their first pregnancy) were more likely to have a child with a secure attachment model at the age of 1 year. In particular, the longitudinal study highlighted that elevated RF in mothers who had suffered from painful and/or traumatic experiences in their childhood was a protective factor against the risk of the child developing insecure and/or disorganized attachment models. On the contrary, these were frequently found in the children of mothers who had suffered traumatic experiences in their own childhood, and who never developed the protective ability to mentalize their own, or their parents’ mental states that were involved in the painful emotional experiences (e.g., severe neglect, loss, physical, or sexual abuse) they experienced (Fonagy et al., 1991). A more recent study (Arnott and Meins, 2007) found that mothers with higher RF showed better mind-mindedness (i.e., the parent’s ability to represent their children’s thoughts and feelings) when their children were 6 months old. In addition, in this study the mothers’ RF predicted child attachment security at 12 months. A later, very recent study (Ensink et al., 2016) replicated the results of the London Parent–Child Project (Fonagy et al., 1991), and found that the RF of the mothers, as assessed during pregnancy, was associated with later adequate parenting as well as infant attachment security.

Afterward, a modified version of the RFS (Slade et al., 2004) was developed to be applied to the Parent Development Interview (PDI) (Aber et al., 1985), a semi-structured interview designed to evaluate the mental representation the parent has of him/herself, as well as of the child, and of their relationship. Studies found that good maternal RF, as assessed in the context of the PDI, mediated the intergenerational transmission of attachment security and was associated with more sensitive and adequate caregiving behavior (Grienenberger et al., 2005; Slade et al., 2005).

A third version of the RFS, i.e., the Child Reflective Functioning Scale (CRFS) was recently developed and validated (Target et al., 2001; Ensink, 2004; Ensink et al., 2015) to be applied to the Child Attachment Interview (CAI) (Shmueli-Goetz et al., 2000). It is a semi-structured interview that was developed to assess attachment models in children aged 7–12. Children with secure attachment showed that higher RF was significantly associated with higher scores on some CAI subscales, namely “Emotional openness” and “Coherence” (Ensink, 2004). A recent study found that maternal RF, as assessed by the PDI, was associated with child RF, and that the latter resulted impaired in children who had experienced sexual abuse (Ensink et al., 2015).

The availability of the CRFS has led to progress in this field, ultimately overcoming some of the previous study limitations. Until recently, the lack of a measure to assess child RF in the context of attachment narratives prevented us from exploring both the impact of mother–child attachment security and the influence of maternal RF on the ability of RF in the child. Previous studies, which focused primarily on preschool aged children, mostly used measures of different components of child mentalization, such as ToM, or emotional understanding in impersonal contexts. These studies found that maternal attachment security predicted the child’s ability to identify painful emotions, to cope with challenging circumstances (Steele et al., 2002), to recognize emotions, especially negative ones (Laible and Thompson, 1998; Steele et al., 1999, 2003, 2008), and to solve false-belief tasks (Fonagy and Target, 1997). Maternal mind-mindedness, as well as maternal RF predicted the child’s performance in ToM tasks as well (Meins et al., 2002, 2003; Steele and Steele, 2008). To date, only few studies have focused on preadolescence (Rosso et al., 2015; Scopesi et al., 2015).

The aim of the current study was to investigate whether, and to what extent, RF during preadolescence is associated with maternal attachment security and RF, and with the child’s attachment security. Based on the available literature, we expected to find an association between child RF, maternal attachment security and RF, and child attachment security even though some studies (de Vito and Muscetta, 1998; Ammaniti et al., 2000; Ammaniti and Sergi, 2003) pointed out that in the transition to adolescence children might more frequently adopt dismissing strategies toward their parents which could decrease their ability to mentalize in the context of their closest familial relationships.

Previous studies also showed that dismissing attachment correlated both with an impairment of the ability to process negative emotions, particularly sadness (Strathearn et al., 2009), and to a proneness to inhibit negative affective responses (Leckman et al., 2004; Strathearn, 2006; Crittenden, 2008). Conversely, it was found that secure mothers showed better attunement with their children and greater ability to repair mismatched states during free play (Riva Crugnola et al., 2013), as well as the maternal proclivity to talk about painful emotions predicted emotional understanding in children (Dunn and Brown, 2001), as well as the early acquisition of ToM (Hughes and Dunn, 2002). Mixed emotional understanding in children was also predicted by their attachment security (Ensink, 2004). Recent studies (Rosso et al., 2015; Scopesi et al., 2015) confirmed the association between a dismissing model and impaired mentalization, as well as the association between the maternal ability to mentalize mixed emotions and mentalization in their children. Thus, the aims of the current study also include investigating (1) whether and to what extent dismissing and preoccupied maternal defensive strategies are associated with an impairment of RF in children, (2) whether the maternal ability to mentalize mixed-ambivalent mental states is associated with higher RF in children. Replication of the findings that were observed in the previous studies was expected. To our knowledge, no empirical studies have ever been conducted to investigate the impact of the preoccupied state of mind on mentalization ability. Fonagy et al. (2010) hypothesized that preoccupied individuals showed strong activation of the attachment system and simultaneous deactivation of the mentalization system. More recently, Fonagy et al. (2016) found that psychologically suffering mothers used mental state talk extensively in their narrative which, however, was not really a marker of authentic mentalization. In line with these hypotheses, we could assume that children of preoccupied mothers do not show good mentalizing, but it would be more cautious in this regard to consider the current study as an exploratory one.

## Materials and Methods

### Participants

Thirty-nine mother–child dyads were recruited on a volunteer basis at an Italian public school. Children were aged 12.3–12.9 years, there were 25 (64.1%) males and 14 (35.9%) females, mostly (74.4%) from intact families. In order to exclude children with physical or psychological impairments, mothers were interviewed regarding the child’s developmental history, while teachers were briefly questioned about learning and/or behavioral disorders. Twenty-three parents (59%) gave consent for their children to be administered the verbal scale of the Wechsler Intelligence Scale for Children (WISC)-III. The Verbal IQ of the children was found to range from 99 to 145 (*M* = 116.96, *SD* = 12.8). Mothers came from working and middle class backgrounds. They were aged between 37 and 53 years (*M* = 42.95; *SD* = 4.36), and their level of education ranged from 8 to 23 years (*M* = 13.31; *SD* = 3.65). All but two were employed.

### Measures

#### Maternal Attachment Models

The AAI (George et al., 1985) was administered to the mothers. It is a semi-structured, hour-long interview designed to classify the state of mind with respect to early attachment experiences. The protocol consists of 18 questions. The interviews begin by asking the subject to describe his/her relationship with their parents during childhood. Then he/she is requested to give five adjectives that describe the relationship with each parent and to recall specific memories that would support the previously chosen adjectives. The next questions ask about the experiences of emotional distress, physical injury, illness and separation from parents during their childhood. The subject is further asked about any possible experiences of rejection, abuse, maltreatment and loss. The interviewee is also asked to give his/her opinion about the impact of their childhood experiences on their personality and the mental states underlying their parents’ behavior. Finally, the interview questions shift to the current relationship with their parents, and the present relationship with their own children, if any. The last question requires them to describe how their experiences of being parented impact on their own parenting. According to the Main and Goldwyn (1998) coding system, the subjects are judged “secure/autonomous” if the narrative is sufficiently coherent regardless of the positive or negative quality of their relationships during childhood. The transcripts are classified as “dismissing” when the speaker shows an attempt to minimize the influence of attachment experiences, in particular idealizing or derogating the attachment figures. The category “preoccupied” is assigned to people who appear entangled in their past experiences. They may be confused, passive, vague, fearful, overwhelmed or angry, conflicted and unconvincingly analytical. “Unresolved/Disorganized” is an additional category that is assigned when the narrative contains markers of lapses in the monitoring of reasoning or discourse during the discussion of experiences of loss and/or abuse. The category “cannot classify” is assigned to those transcripts that show a mixture of inconsistent and incompatible states of mind. In the non-clinical populations the latter classification is rarely assigned. According to the findings of the most recent meta-analysis, the following distribution was observed in the non-clinical adult population: 58% secure, 23% dismissing, 19% preoccupied, and 18% additionally classified unresolved/disorganized (Bakermans-Kranenburg and van IJzendoorn, 2009). Several studies have supported the power of the AAI to predict parenting and subsequent infant–parent attachment (Fonagy et al., 1991; van IJzendoorn, 1995; Bakermans-Kranenburg and van IJzendoorn, 2009; Berthelot et al., 2015).

In the current study, the two-way classification (Secure vs. Insecure) was used. The decision to dichotomize the sample was the only choice since, due to the limited number of participants in the study, our sample included only 15 mothers who were classified as Insecure (five Dismissing, seven Preoccupied, and three Unresolved). Furthermore, a dimensional approach to the AAI was also utilized, as suggested by recent studies (Bakermans-Kranenburg and van IJzendoorn, 2009; Whipple et al., 2011) after Roisman et al. (2007) explored the AAI latent structure and found two dimensions, namely the dismissing and the preoccupied dimension. Using the state of mind scales in the analyses is also recommended because it allows to investigate the impact of the dismissing and the preoccupied dimensions with enhanced statistical power (Roisman et al., 2007). Thus, we considered the subscales “Idealization regarding mother,” “Idealization regarding father,” “Overall derogation of attachment,” and “Coherence of the mind” to explore the definite impact of the dismissing strategies and the subscales “Passivity,” “Involving anger toward mother,” and “Involving anger toward father” to investigate the influence of the maternal preoccupied state of the mind on the children’s RF. All of the AAIs were coded in terms of the Berkeley AAI System (Main and Goldwyn, 1998) by a licensed coder, blinded to scores on other measures. Eight transcripts (20%) were then randomly selected and re-coded by the first author. The resulting inter-rater reliability was satisfactory (Cohen’s *k* = 0.86 for overall classification, and *ICC* ranging from 0.81 to 0.85 for the subscales).

#### Maternal Reflective Functioning

The Adult Reflective Functioning Scale (ARFS) (Fonagy et al., 1998) was applied to the AAI transcripts to evaluate maternal RF. In coding RF, some AAI questions are considered “Demand Questions” in that they require RF (e.g., “Why do you think your parents behaved as they did during your childhood?”), while other questions are called “Permit Questions” in that they do not require, but only allow RF (e.g., “Could you describe your first separation from your parents?”). According to the scoring guidelines, the following four markers of RF are identified: “Awareness of the nature of mental states” (marker A), “Explicit effort to tease out mental states underlying behavior” (marker B), “Recognizing developmental aspects of mental states” (Marker C), and “Mental states in relation to the interviewer” (Marker D). After rating each identified passage of the AAI, an overall classification is assigned to the interview as a whole, ranging from -1 (negative RF) to 9 (exceptional RF). In this study, in addition to the overall RFS rating score, we considered three further RF variables on the basis of a recent study (Rosso et al., 2015), namely the frequency of RF in the context of positive, negative, and mixed-ambivalent mental states (e.g., “I felt secure with my mum, because she always tried to comfort me”; “Unfortunately, I often got mad at my mother, it seemed that she could not understand me when I was sad”; “I really don’t know how the relationship with my mother was when I was a child, sometimes I felt well with her, sometimes I felt some kind of irritation, maybe I was really sensitive to her sudden mood swings, without understanding that she was terribly depressed”). Validation studies of RFS (Fonagy et al., 1998) showed discriminant and predictive validity, good inter-rater reliability, low correlation with education level, and no correlation with socioeconomic status (SES) or age.

In the present study, the RF score did not correlate to either the mothers’ level of education (*r* = -0.032, *p* = 0.845) or to the mothers’ age (*r* = 0.121, *p* = 0.463). The first author, who was blinded to the scores on the other measures, rated the transcripts according to the guidelines manual (Fonagy et al., 1998), then eight transcripts (20%) were randomly selected and re-coded by an independent coder. The resulting inter-rater reliability was excellent (*ICC* = 0.82).

#### Child Attachment Models

The CAI (Shmueli-Goetz et al., 2000, 2008, 2011; Target et al., 2003) was administered to the children. It is a measure designed to assess attachment models in children from 7 to 12 years of age. The protocol includes 19 questions about the composition of the family, and about the child him/herself and the relationship with his/her parents. The child is encouraged to talk about specific relationship episodes involving each parent, even concerning moments in which he/she was ill or felt troubled or was in conflict with them or in need of help. Similarly to the AAI, the CAI investigates the emotional reactions to experiences of mourning as well as of separations. Coding the protocol takes into account not only an analysis of the speech, but also the non-verbal behavior of the child. A score ranging from -1 to 9 is assigned to the following subscales: “Emotional openness,” “Balance of positive/negative references to attachment figures,” “Use of examples,” “Preoccupied involving anger,” “Idealization,” “Dismissal of attachment,” “Resolution of conflict,” “Atypical/Disorganized behavior,” and “Overall coherence.” Then, a main attachment classification (Secure, Dismissing, Preoccupied, Disorganized) is assigned individually to the mother and to the father. Secure children show greater ability to express and to identify emotions and to give examples, as well as low levels of anger, idealization, dismissal/derogation of attachment, a higher balance of positive and negative references, and the ability to resolve conflicts constructively. Preoccupied children are entangled in their painful experiences, sometimes overwhelmed by anger feelings, and excessively focused on the parent. Dismissing children are highly rated on “Idealization” and/or “Dismissal,” as well as low rated on “Emotional openness” “Balance of positive/negative references to attachment figures,” “Use of examples,” “Resolution of conflict,” and “Overall coherence.” Disorganized children often show a proclivity to control through punitive or care-giving behavior. During the interview, these children may show sudden changes in the affective tone, interruptions in speech, affective inadequacy, and/or bizarre behavior. In some cases, they exhibit unrealistic self-representations. CAI validation studies (Target et al., 2003; Shmueli-Goetz et al., 2008; Venta et al., 2014; Borelli et al., 2016) conducted on clinical and non-clinical populations showed good psychometric properties. Inter-rater reliability was good (*k* between 0.58 and 0.93), both between expert coders and between students who had received 3 days of training. The distribution of attachment classifications in non-clinical samples was in line with what is reported in the literature (i.e., 66% secure, 30% dismissing, 4% preoccupied with respect to the mother, and 64% secure, 30% dismissing, and 6% preoccupied with respect to the father). The concordance of classifications between mother and father was very high (92%, *k* = 0.84). The group of scales related to the state of mind showed a high internal consistency (Cronbach’sα = 0.87). The test-retest reliability showed *k* values between 0.52 and 0.81 after 3 months, and *k* values between 0.52 and 0.74 after 1 year. The classification with respect to the mother showed higher temporal stability compared to the classification toward the father. No significant differences were observed when comparing secure and insecure children, with regard to age, gender, SES, ethnicity and verbal IQ. A significant association was instead observed between attachment classification of the children and that of their mothers (Shmueli-Goetz et al., 2008).

In the current study, the second author, who was blinded to scores on other measures, rated the transcripts according to the guidelines (Shmueli-Goetz et al., 2011), then eight transcripts (20%) were randomly selected and re-coded by an independent coder. The resulting inter-rater reliability was excellent (*k* = 0.88 for the overall classification and *ICCs* ranging from 0.84 to 0.88 for subscales).

#### Child Reflective Functioning

The CRFS (Target et al., 2001) was developed on the conceptual basis of the ARFS, with modifications to the guidelines so as to apply it to children. As for AAI, the markers of RF include “Awareness of qualities of mental states,” “Explicit effort to tease out mental states underlying behavior,” “Recognizing that mental states develop in the context of developmental, psychobiological, and social processes,” and “Mental states in relation to the interviewer.” It must be kept in mind that as compared to adults, children often give evidence of RF in more implicit ways, for example by mimicking, changing their tone of voice and by facial expressions. This is why coding from videotaped interviews is also needed since coding from transcripts alone is not enough. CRFS inter-rater reliability was found to be good, with ICC ranging from 0.60 to 1.00, with a median of 0.93, temporal stability was found to be high over a 3-month period and adequate over 12 months (Ensink, 2004). A recent study (Ensink et al., 2015) supported the validity of the CRFS in distinguishing sexually abused children from a community control group.

In this study, in addition to the overall CRFS rating score, we considered the frequency of RF in the context of positive, negative, and mixed-ambivalent mental states, just as we did when coding RF in the mothers. All the CAI transcripts were coded according to the CRFS guidelines (Target et al., 2001) by a licensed coder, blinded to scores on the other measures. Then 8 transcripts (20%) were randomly selected and re-coded by the first author. The resulting inter-rater reliability was satisfactory (*ICC* = 0.85).

#### Children’s Verbal Intelligence

The WISC-III verbal scale was administered to assess the children’s verbal IQ.

### Procedure

Mothers and children agreed to participate in this study after receiving a letter from the headmaster of the school attended by the children. The letter presented our research project as a study aimed at investigating the inter-generational transmission of attachment models. Only 13% of the families of children attending the second year of the middle school agreed to be contacted further. Mothers had a brief interview with the researchers aimed at further illustrating the study and at collecting the developmental history of the children to rule out physical or mental disorders, after which the children were informed about the aim of the study. All of the contacted children agreed to participate, then both parents gave their written consent. While all of them gave their consent for the interviews, only 23 families gave their consent to administer the WISC-III verbal scale. Graduate psychology students, who had previously been trained by the first author in the administration of the AAI and the CAI, administered the interviews in rooms made available by the headmaster inside the school. The AAIs were audiotaped, the CAIs were videotaped, and then both were transcribed verbatim. All the coders involved in the study had received their coding license after ad hoc training at the Anna Freud Centre and University College in London. The study followed the 2010 ethical guidelines of the APA (American Psychological Association, 2010).

## Results

### Preliminary Analyses

First of all, we checked the distribution of the variables of interest. All, but maternal derogation, maternal involving anger toward mother and father, and maternal references to mixed-ambivalent mental states, resulted normally distributed. Thus, in the subsequent analyses non-parametric statistics were used only for the four not normally distributed variables. Then, we explored the data for possibly puzzling variables. Gender differences in CRF-overall score, *F*(1,38) = 0.342, *p* > 0.05, CRF-references to positive mental states, *F*(1,38) = 0.172, *p* > 0.05, CRF-references to negative mental states, *F*(1,38) = 0.064, *p* > 0.05, and CRF-references to mixed-ambivalent mental states, *F*(1,38) = 2.152, *p* > 0.05, were not significant. No significant correlation emerged between maternal level of education, maternal RF (*r* = -0.032), and child RF (*r* = 0.206). The children’s verbal I.Q. did not correlate with child RF (*r* = 0.062).

### Child Reflective Functioning and Maternal Attachment

According to the AAI coding system, 24 mothers were classified as secure and 15 mothers as insecure. The children of secure and insecure mothers were compared on RFS scores using independent *t*-test. A moderate effect of the group (Cohen’s *d* = 0.63) was found regarding overall CRFS, with higher scores being observed in the children of secure mothers. Comparisons are reported in **Table 1**.

**Table 1:** Child Reflective Functioning Scale (CRFS) descriptive statistics and group comparisons between children of secure and insecure mothers.

	Secure mothers *N* = 24		Insecure mothers *N* = 15		Comparisons		
Variable	*M*	*SD*	*M*	*SD*	*t*	*p*	*d*
CRFS	3.92	1.47	3.13	0.99	-1.816	0.078	0.64
CPMS	4.46	3.98	4.27	3.26	-0.156	0.877	0.05
CNMS	8.96	4.75	10.13	5.15	0.728	0.471	-0.24
CMMS	2.42	2.24	2.66	1.72	0.369	0.715	-0.12

*M, mean; SD, standard deviation; t, *t*-statistic; p, *p*-value; d, Cohen’s measure of effect size (|d| < 0.20: negligible; |0.20| < d < |0.50| : small; |0.50| < d < |0.80| moderate; d > |0.80| : large); CRFS, Children’s overall Score reported on Child Reflective Functioning Scale; CPMS, Children’s references to positive mental states in the context of RF; CNMS, Children’s references to negative mental states in the context of RF; CMMS, Children’s references to mixed-ambivalent mental states in the context of RF.*

Correlation analysis was used to investigate the association between the maternal scales of mind referred to attachment and the child RF. The results are shown in **Table 2**.

**Table 2:** Descriptive statistics for maternal scores on AAI subscales and correlations between maternal AAI subscales and child reflective functioning scores.

	*M*	*SD*	CRFS	CPMM	CNMS	CMMS
Maternal idealization M	2.40	1.61	-0.085	0.147	0.138	0.025
Maternal idealization F	2.35	1.51	-0.101	-0.044	0.197	0.035
Maternal overall derogation	2.10	1.97	-0.327ˆ*	-0.220	0.036	-0.167
Maternal passivity	3.01	1.92	-0.161	0.009	0.187	0.056
Maternal involving anger M	2.10	2.31	-0.252	-0.206	0.012	-0.050
Maternal involving anger F	1.59	1.70	-0.120	0.044	-0.161	0.063
Maternal coherence of mind	5.69	1.55	0.326ˆ*	0.049	0.015	0.081

*M, mean; SD, standard deviation; CRFS, Children’s overall score reported on Child Reflective Functioning Scale; CPMS, Children’s references to positive mental states in the context of RF; CNMS, Children’s references to negative mental states in the context of RF; CMMS, Children’s references to mixed-ambivalent mental states in the context of RF; Maternal Idealization M, maternal score on “Idealization toward mother” AAI subscale; Maternal Idealization F, maternal score on “Idealization toward father” AAI subscale; Maternal Overall Derogation, maternal score on “Overall Derogation” AAI subscale; Maternal Passivity, maternal scores on “Passivity” AAI subscale; Maternal Involving Anger M, maternal score on “Involving anger toward mother” AAI subscale; Maternal Involving Anger F, maternal score on “Involving anger toward father” AAI subscale; Maternal Coherence of Mind, maternal score on “Coherence of Mind” AAI subscale; ^∗^*p* < 0.05.*

Child RF correlated significantly positively with maternal Coherence of Mind (*r* = 0.326, *p* = 0.043) and negatively with Maternal overall derogation of attachment (ρ = -0.327, *p* = 0.043). No significant associations emerged between child RF and the maternal idealization of her relationships with her parents during her childhood. A negative association (ρ = -0.252), albeit not statistically significant, was found between maternal “Involving anger toward mother” and child RF.

### Child Reflective Functioning and Child Attachment

Twenty-two children (56.4%) were classified as secure toward their mother, and 17 children were rated as insecure, of whom 14 (35.9%) were dismissing and three (7.7%) were rated as preoccupied. None of the children were classified as disorganized. Secure children obtained higher scores on CRFS (*M* = 4.14, *SD* = 1.36) compared to insecure children (*M* = 2.94; *SD* = 1.03). The comparison was carried out using the independent *t*-test and yielded a significant difference between the two groups (*t* = -3.021, *p* = 0.005) as well as a large effect size (Cohen’s *d* = 0.99).

Correlation analysis was used to explore the association between the scores obtained on the CAI subscales and the CRFS. Results are provided in **Table 3**.

**Table 3:** Descriptive statistics for CAI subscales and correlations between CAI subscales and child reflective functioning scores.

	*M*	*SD*	CRFS	CPMM	CNMS	CMMS
Emotional openness	5.06	2.10	0.607***	0.402*	0.469**	0.416**
Balance of references to AF’s	5.09	1.68	0.382*	0.248	0.208	0.217
Use of examples	5.04	2.10	0.552***	0.378*	0.445**	0.385*
Anger toward mother	1.40	.97	0.149	-0.022	0.134	0.186
Anger toward father	1.58	1.22	0.043	-0.054	0.258	-0.043
Idealization of mother	3.10	1.78	-0.208	0.036	-0.111	-0.109
Idealization of father	2.71	1.67	-0.350*	-0.045	-0.313	-0.117
Dismissal of mother	2.77	2.02	-0.458**	-0.466**	-0.474**	-0.431**
Dismissal of father	3.01	2.11	-0.423**	-0.416**	-0.360*	-0.369*
Resolution of conflicts	5.53	1.26	0.472**	0.269	0.151	0.175
Overall coherence	5.28	1.77	0.549***	0.287	0.352*	0.898***

*M, mean; SD, standard deviation; CRFS, Children’s overall (ccore)elim score reported on Child Reflective Functioning Scale; CPMS, Children’s references to positive mental states in the context of RF; CNMS, Children’s references to negative mental states in the context of RF; CMMS, Children’s references to mixed-ambivalent mental states in the context of RF; ^∗^*p* < 0.05; ^∗∗^*p* < 0.01; ^∗∗∗^*p* < 0.001.*

Overall CRFS score correlated significantly with “Emotional openness” (*r* = 0.607), “Balance of references to Attachment Figures” (*r* = 0.382), “Use of examples” (*r* = 0.552), “Resolution of conflicts” (*r* = 0.472), and “Overall coherence” (*r* = 0.549). An inverse correlation was observed between Overall CRFS score and “Idealization of father”(*r* = -0.350), “Dismissal of mother” (*r* = -0.458), and “Dismissal of father” (*r* = -0.423). The children’s ability to mentalize positive mental states was significantly positively associated with “Emotional openness” (*r* = 0.402), “Use of examples” (*r* = 0.378), and significantly negatively associated with “Dismissal of mother” (*r* = -0.466), and “Dismissal of father” (*r* = -0.416). The children’s ability to mentalize negative mental states was significantly positively associated with “Emotional openness” (*r* = 0.469), “Use of examples” (*r* = 0.445), and “Overall coherence” (*r* = 0.352), whereas it was significantly negatively associated with “Dismissal of mother” (*r* = -0.474), and “Dismissal of father” (*r* = -0.360). The ability of the children to mentalize mixed-ambivalent mental states also resulted significantly positively associated with “Emotional openness” (*r* = 0.416), “Use of examples” (*r* = 0.385), and “Overall coherence” (*r* = 0.898), whereas it was significantly negatively associated with “Dismissal of mother” (*r* = -0.431), and “Dismissal of father” (*r* = -0.369).

### Child Reflective Functioning and Maternal Reflective Functioning

Correlation analysis was also conducted to investigate the association of child RF with maternal RF. As reported in **Table 4**, a positive significant association emerged between child and maternal overall scores on RFS (*r* = 0.375). In particular, the children’s overall RF score was associated with maternal ability to mentalize negative mental states (*r* = 0.348), as well as maternal ability to mentalize mixed-ambivalent mental states (ρ = 0.508). The maternal ability to mentalize mixed-ambivalent mental states was also significantly associated with the children’s ability to mentalize positive (ρ = 0.325), negative (ρ = 0.426), and mixed-ambivalent (ρ = 0.434) mental states.

**Table 4:** Correlations between maternal and child reflective functioning scores.

	CRFS	CPMS	CNMS	CMMS
MRFS	0.375*	0.055	0.094	0.179
MPMS	0.168	-0.001	-0.216	-0.044
MNMS	0.348*	0.055	0.183	0.081
MMMS	0.508**	0.325*	0.426**	0.434**

*CRFS, Children’s overall score reported on Child Reflective Functioning Scale; CPMS, Children’s references to positive mental states in the context of RF; CNMS, Children’s references to negative mental states in the context of RF; CMMS, Children’s references to mixed-ambivalent mental states in the context of RF; MRFS, Mothers’ Overall Score reported on Reflective Functioning Scale; MPMS, Mothers’ references to positive mental states in the context of RF; MNMS, Mothers’ references to negative mental states in the context of RF; MMMS, Mothers’ references to mixed-ambivalent mental states in the context of RF; ^∗^*p* < 0.05; ^∗∗^*p* < 0.01.*

To explore the extent to which maternal security of attachment and maternal RF might predict RF in children, a stepwise regression analysis was performed using maternal “Coherence of mind,” maternal overall RFS score, and maternal references to mixed-ambivalent mental states as predictors of the children’s RF. The final models are shown in **Table 5**. The models account for approximately 21% of the variance in children’s overall RF score, and about 22% of the variance in children’s references to mixed-ambivalent mental states. Specifically, only maternal overall RFS score predicted children’s overall RFS score (*t* = 3.082, *p* = 0.004), and only maternal ability to mentalize in mixed-ambivalent mental states predicted the corresponding ability in the children (*t* = 3.167, *p* = 0.003).

**Table 5:** Stepwise regression analyses for predicting child reflective functioning.

	Children’s overall RF score *F*(1,36) = 9.497; *R*^2^= 0.209; *p* = 0.004						Children’s references to MMS *F*(1,36) = 10.029; *R*^2^= 0.218; *p* = 0.003				
	*B*	*SE*	β	*t*	*p*		*B*	*SE*	β	*t*	*p*
**MRFS**	**0.453**	**0.147**	**0.457**	**3.082**	**0.004**	**MMMS**	**0.416**	**0.131**	**0.467**	**3.167**	**0.003**
CoM	0.128	0.206	0.141	0.622	0.538	CoM	0.064	0.315	0.047	0.204	0.840
MMMS	0.138	0.113	0.234	1.226	0.229	MRFS	-0.142	0.406	-0.095	-0.351	0.728

*Final model in bold; MMS, mixed-ambivalent mental states; MRFS, Mothers’ Overall Score reported on Reflective Functioning Scale; CoM, Maternal Coherence of Mind; MMMS, mothers’ references to mixed-ambivalent mental states in the context of RF.*

## Discussion

### Child Reflective Functioning and Maternal Attachment

The children of mothers who showed a secure attachment model regarding the relationship with their own parents during their childhood reported higher levels of RF than did the children of mothers who were classified as insecure on the AAI. Child RF was positively associated with maternal “Coherence of the Mind” on the AAI and negatively associated with maternal derogation of attachment. No association was found between Child RF and maternal idealizing strategies in the context of the AAI. A negative association, albeit not statistically significant, was found between maternal “Involving anger toward mother” and child RF.

These findings were mostly consistent with our hypotheses, and replicated results from previous studies. Thus, support was given to the notion that the maternal coherent mental representation of her personal history, free from rigid defensive strategies, both maximizing and minimizing the importance of attachment relationships, allows the mother to freely access and process emotions in herself as well as in her child, in turn promoting the child’s RF. Previous studies already found that securely attached mothers showed more emotional openness, whereas dismissing mothers were prone to minimize internalizing emotions in themselves as well as in their children, specifically by not being responsive to emotions of fear and sadness in their children (DeOliveira et al., 2005). The ability to accurately identify the child’s emotions and to understand the causes of his/her distress was found to be related to attachment security, while experiences of neglect in childhood were found to be associated with an impairment of this maternal ability. Insecure women were less accurate in identifying emotions in children, and were more prone to negative attributions, and to be amused or neutral in the face of the child’s distress (Leerkes and Siepak, 2006). In line with these findings, the results of the current study confirm that maternal derogation of attachment is specifically associated with impaired RF in children. A mother who derogates her emotional and attachment needs may be unable to be sympathetic with her child’s emotional needs, and it could be argued that her empathetic deficit in turn weakens her child’s ability to recognize, to pay attention to, and to place importance on mental states. It has been found that the maternal proneness to contemplate children’s negative emotions predicted emotional understanding in children (Dunn and Brown, 2001) whereas maternal difficulties in understanding the child’s mind predicted an impairment in the children’s ability to identify and deal with negative emotions (Sharp et al., 2006).

It was noteworthy that the results of the current study highlighted that maternal derogation, rather than maternal idealization, was associated with the child’s impairment in RF. We could assume that idealizing strategies have a less destructive influence on mentalization, possibly impairing hostile feelings toward their attachment figures rather than impairing their entire emotional awareness, and thereby damaging RF to a lesser degree. This finding suggests that the overall dismissing category might be confusing in that it includes different sub-classifications: DS1 and DS3 (based mostly on the idealizing strategy), and DS2 (based on the derogating strategy). Results from the current study suggest that it is the maternal dismissing strategy based on derogation of the attachment figures as well as of one’s own attachment needs that has a more disruptive impact on the child’s mentalization.

### Child Reflective Functioning and Attachment Security

A highly significant association was also found between child attachment security and child RF, thus replicating the results of the CRFS validation study (Ensink, 2004). Children who were rated as being more emotionally open, more able to balance positive and negative descriptions of their parents, more prone to support their assertions through examples, and more able to positively resolve conflicts with their parents showed better RF. On the contrary, children who more often resorted to idealization and dismissal toward their parents showed a lesser degree of RF. Moreover, it is remarkable that the child’s dismissal strategy and not the child’s idealizing strategy negatively correlated with the child’s RF. Yet, findings from the current study highlighted the more disruptive influence of the dismissal strategy on the mentalizing ability. Notably, a very strong association was found between the score on the “Overall coherence” subscale and the child’s ability to mentalize mixed-ambivalent mental states in the context of their family relationships. Thus, these results strongly support the definite relationships that exist between attachment security and RF in the context of family relationships. Fonagy et al. (2010) recently reported specific associations between different attachment models and responses to the activation of the attachment system. Whereas secure individuals were able to maintain the mentalization and attachment systems simultaneously, dismissing individuals did not activate the attachment system, and preoccupied individuals showed strong activation of the attachment system and simultaneous deactivation of the mentalization system. Early studies assumed that since secure children feel an inner sense of emotional security in their relationship with their parents, they do not activate an attachment system and therefore are able to maintain an active mentalization system (Fonagy, 2006; Fonagy and Target, 2008). However, it was more recently hypothesized (Fonagy et al., 2010) that maternal mentalization mediated the relationship between secure attachment and mentalization in children.

### Child and Maternal Reflective Functioning

As expected, child and maternal RF resulted significantly positively correlated with each other. Correlation analysis yielded interesting findings showing that, above all, maternal ability to mentalize negative as well as mixed-ambivalent mental states correlated with the child RF. In particular, only maternal RF (and not maternal attachment security) predicted child RF, and only maternal ability to mentalize mixed-ambivalent mental states predicted the corresponding ability in the children. Thus, results from the present study add support to the hypothesis according to which maternal mentalization, more than maternal attachment security promotes mentalizing ability in children.

According to Fonagy et al. (2010), the maternal ability not to be overwhelmed by the emotional experiences of the child, especially when they are intense and/or painful, and her ability to mirror them in a marked and contingent way (Gergely and Watson, 1996), enhance the child’s ability to effectively regulate emotions, allowing him/her to keep both attachment and mentalization systems activated. On the basis of this hypothesis, emotional regulation, rather than secure attachment, would allow mentalization. In other words, effective emotional regulation, promoted by a mother who is able to mentalize even in conditions of increased arousal as well as in the context of negative and ambivalent mental states, mediates the relationship between attachment security and mentalization ability. Results of the current study seem to support the hypothesis put forth by Fonagy et al. (2007). They argued that particularly negative affects related to inevitable conflicts (provided they were moderate and experienced in the context of a good enough relationship) elicit the emergence of mentalization. At the same time, a good enough mother–child relationship provides the necessary emotional containment to promote the ability to mentalize. Our study suggests that mothers who are open to recognizing the emotional experience related to mixed-ambivalent mental states both in themselves and in their children, and to reflect upon it without being overwhelmed or in need to deny or to avoid it, are more able to promote the corresponding mentalizing ability in their children. However, further studies are needed to investigate whether and to what extent mothers with better mentalizing abilities use more mental state talk in the conversations with their children, and whether and to what extent the maternal ability of mental state talk mediates the intergenerational transmission of RF.

Furthermore, findings from the current study provide a fresh contribution to the research in this field, in that previous studies investigated the relationship between maternal and child mentalization comparing indeed different components (e.g., mind-mindedness, emotional understanding, ToM, mental-state talk) of the multifaceted construct of mentalization in mothers and in children. To the best of our knowledge, this is the first study to compare the same operationalization of mentalization, namely RF, in mothers and their children by using the narratives about attachment in close family relationships both for mothers and for children. A previous study (Ensink et al., 2015) investigated RF in mothers and children of about 10 years of age on average, by assessing maternal RF in the context of the PDI. Ensink’s study differs from ours because in that context the authors specifically evaluated the maternal ability to mentalize the child, instead of mentalizing the mother’s own mental representations of her early attachment relationships. As Ensink et al. (2016) stated, taking into account the maternal RF even in the AAI, and not only in the PDI, is crucial because the mother’s mentalization regarding her attachment experiences in childhood plays a critical role in her parenting. Maternal RF about her personal attachment history helps the mother to put herself in her child’s shoes and be interested in his/her emotional experience and mental states. In addition, maternal RF might help the mother to understand what impact her feelings and thoughts could have on the child, thus preventing negative parenting.

Furthermore, the present work contributes to the study of the intergenerational transmission of RF in preadolescence, a rarely investigated developmental phase with regard to mentalization. As expected, we found a slightly increased frequency of the dismissing model in preadolescents. This finding, which is in line with previous studies (e.g., Weinfield et al., 2004; Doyle et al., 2009), could raise questions about the generalizability of the results of the current study. However, it is noteworthy that a significant association was observed between child and maternal RF, even in this developmental stage in which children are usually striving to achieve more autonomy.

The relatively small sample size (due in part to the very time consuming measures of attachment model and RF) prevented us from investigation the association between the distinct models of insecure attachment, namely dismissing, preoccupied, and disorganized, and distinct impairment of RF. Lastly, in addition to the above mentioned limitations of the study, it should be pointed out that only a very small number of the contacted families agreed to participate in the study. On the one hand, this was expected because of the very confidential and intimate nature of the measures that were used, on the other hand it might be questioned whether and to what extent the sample could be considered representative of the population.

## Conclusion

The present study investigated whether, and to what extent, RF during preadolescence was associated with maternal attachment security and RF, and with the child’s attachment security. Results yielded significantly positive associations between child RF, maternal attachment security, maternal RF as well as child attachment security. On the contrary, maternal derogation of attachment and children’s dismissing strategies were associated with lower RF in children. Specifically, only maternal RF (and not maternal attachment security) predicted child RF, and only maternal ability to mentalize mixed-ambivalent mental states predicted the corresponding ability in the children.

## Author Contributions

AR designed the study, coordinated data collection, performed the statistical analyses and prepared the first draft of the article. CA contributed to the search for references, coded the CAI transcripts, cooperated in performing the statistical analyses, and contributed to the final version.

## Conflict of Interest Statement

The authors declare that the research was conducted in the absence of any commercial or financial relationships that could be construed as a potential conflict of interest.
